# Impact of Cancer Type and Treatment Protocol on Cardiac Function in Pediatric Oncology Patients: An Analysis Utilizing Speckle Tracking, Global Longitudinal Strain, and Myocardial Performance Index

**DOI:** 10.3390/diagnostics13172830

**Published:** 2023-08-31

**Authors:** Andrada Mara Ardelean, Ioana Cristina Olariu, Raluca Isac, Akhila Nalla, Ruxandra Jurac, Cristiana Stolojanu, Mircea Murariu, Roxana Manuela Fericean, Laurentiu Braescu, Adelina Mavrea, Catalin Dumitru, Gabriela Doros

**Affiliations:** 1 Department of Pediatrics, “Victor Babes” University of Medicine and Pharmacy, Eftimie Murgu Square 2, 300041 Timisoara, Romania; andrada.micsescu-olah@umft.ro (A.M.A.); olariu.cristina@umft.ro (I.C.O.); isac.raluca@umft.ro (R.I.); steflea.ruxandra@umft.ro (R.J.); gdoros@gmail.com (G.D.); 2 Doctoral School, “Victor Babes” University of Medicine and Pharmacy, Eftimie Murgu Square 2, 300041 Timisoara, Romania; stolojanu.cristiana@umft.ro (C.S.); mircea.1192@yahoo.com (M.M.); manuela.fericean@umft.ro (R.M.F.); braescu.laurentiu@umft.ro (L.B.); 3 Louis Turcanu Emergency Hospital for Children, Iosif Nemoianu Street 2, 300011 Timișoara, Romania; 4 Department of General Medicine, MNR Medical College, Sangareddy 502294, Telangana, India; akhila.nalla@gmail.com; 5 Methodological and Infectious Diseases Research Center, Department of Infectious Diseases, “Victor Babes” University of Medicine and Pharmacy, 300041 Timisoara, Romania; 6 Department of Cardiovascular Surgery, “Victor Babes” University of Medicine and Pharmacy, Eftimie Murgu Square 2, 300041 Timisoara, Romania; 7 Center for Translational Research and Systems Medicine (CERT-MEDS), “Victor Babes” University of Medicine and Pharmacy, Eftimie Murgu Square 2, 300041 Timisoara, Romania; 8 Department of Internal Medicine I, Cardiology Clinic, “Victor Babes” University of Medicine and Pharmacy Timisoara, Eftimie Murgu Square 2, 300041 Timisoara, Romania; 9 Department of Obstetrics and Gynecology, “Victor Babes” University of Medicine and Pharmacy Timisoara, 300041 Timisoara, Romania; dumitru.catalin@umft.ro

**Keywords:** pediatrics, oncology, chemotherapy, cardiac function tests

## Abstract

Pediatric hemato-oncology patients undergoing anthracycline therapy are at risk of cardiotoxicity, with disease type and treatment intensity potentially affecting cardiac function. Novel echocardiographic measures like speckle tracking echocardiography (STE), global longitudinal strain (GLS), and the myocardial performance index (MPI) may predict early changes in cardiac function not detected by traditional methods. This study aimed to assess the impact of cancer type and treatment protocol on these parameters and their potential in predicting long-term cardiac complications. We conducted a single-center, retrospective cohort study of 99 pediatric oncology patients and 46 controls that were assessed at 3, 6, and 12 months. The median age was 10.7 ± 4.4 years for cases and 10.2 ± 3.6 years for controls. STE, GLS, and MPI were measured, and statistical analyses were performed to determine any significant correlations with cardiotoxicity. Significant variations were observed in traditional cardiac function measurements between the patient and control groups, with a lower average ejection fraction (EF) of 62.8 ± 5.7% in patients vs. 66.4 ± 6.1% in controls (*p* < 0.001), poorer GLS of −16.3 ± 5.1 in patients compared to −19.0 ± 5.4 in controls (*p* = 0.004), and higher MPI values of 0.37 ± 0.06 in patients compared to 0.55 ± 0.10 in controls, indicating worse overall cardiac function (*p* < 0.001). However, differences in cardiac function measurements by cancer histology or treatment protocol were not statistically significant. Regression analyses showed that the combination of GLS, SMOD, and MPI increased the odds of cardiac toxicity with an odds ratio of 7.30 (95% CI: 2.65–12.81, *p* < 0.001). The study underscores the predictive value of the combined GLS, SMOD, and MPI measurements in pediatric oncology patients undergoing anthracycline treatment for cardiotoxicity. Although variations across cancer types and treatment protocols were not significant, the study emphasizes the potential utility of these novel echocardiographic measures in early detection and long-term prediction of anthracycline-induced cardiotoxicity. Further studies in larger, multi-center cohorts are required for validation.

## 1. Introduction

The continued advancements in pediatric oncology have led to a steady increase in survival rates, providing a truly encouraging trend [1]. Despite this promising development, the life-saving interventions, which often include aggressive chemotherapy and radiotherapy, often cause considerable long-term morbidity [2,3]. One of the most significant and alarming late effects is cardiotoxicity [4]. It is well established that cancer treatments, particularly anthracyclines and chest irradiation, can lead to various cardiac complications such as cardiomyopathy, ischemic heart disease, pericarditis, arrythmia, and congestive heart failure [5]. Furthermore, cardiac damage can occur years or even decades after the completion of therapy, adding an extra dimension to this already complex issue [6].

Traditionally, cardiac function in pediatric oncology patients has been monitored using conventional echocardiographic methods such as ejection fraction (EF) and fractional shortening (FS) [7]. However, these measures have significant limitations, since they are influenced by several factors, including loading conditions and heart rate, and may not accurately represent true myocardial function [8]. Moreover, significant reductions in EF and FS often occur late, when irreversible myocardial damage has already occurred [9]. Therefore, there is an urgent need to improve the early detection of cardiotoxicity in this population.

The damaging effects of treatments like anthracyclines on the heart cannot be understated. Anthracyclines are known to induce cardiotoxicity, a significant side effect that can severely affect the quality of life and overall survival of cancer patients, independent of the oncological prognosis [10]. This cardiotoxicity usually starts with myocardial cell injury, progresses to reductions in left ventricular ejection fraction (LVEF), and, if undetected and untreated, can eventually lead to symptomatic heart failure [10]. Specifically, anthracycline-induced cardiotoxicity can manifest even in a subclinical phase, making early detection paramount. Traditional measures like LVEF may not suffice in capturing these early changes. Advanced echocardiographic techniques, such as 2D Speckle tracking echocardiography (STE), have shown the capability of detecting early myocardial changes, especially during chemotherapy [11,12]. Peak systolic global longitudinal strain, a parameter derived from STE, stands out as an especially consistent indicator of early myocardial damage. Thus, the use of STE, GLS, and MPI can be pivotal, not just in detecting these changes, but also in driving the implementation of cardioprotective treatments before irreversible damage ensues.

In recent years, speckle tracking echocardiography (STE), global longitudinal strain (GLS), and the myocardial performance index (MPI) have emerged as promising tools for the evaluation of myocardial function [13,14]. STE is an angle-independent imaging technique that provides a detailed assessment of myocardial deformation [15]. GLS measures the degree of deformation of the myocardium longitudinally and is considered more sensitive and specific for the early detection of myocardial damage than traditional echocardiographic methods [16]. MPI, also known as the Tei index, is a Doppler-derived index that evaluates both systolic and diastolic cardiac function [17]. These innovative techniques may enable us to detect subtle changes in cardiac function and improve patient outcomes.

However, while these novel methods show great promise, there are still many unknowns regarding their utility in pediatric oncology patients [18,19]. Previous studies have been limited by small sample sizes, heterogeneous patient populations, and lack of long-term follow-up data. Moreover, there is limited research on the potential impact of different disease types and treatment intensity on the results of these measures. In addition, it is unclear whether these measures can predict long-term cardiac outcomes. Early detection of cardiotoxicity is crucial, but it is equally important to understand the implications of these findings. If changes in cardiac function were identified at an early stage, it would be possible to intervene and alter the course of the disease. However, to do this effectively, there is a need to understand how these early changes are associated with long-term outcomes.

Therefore, the hypotheses of the present study are that disease type and treatment intensity have a significant impact on cardiac function as measured by speckle tracking, global longitudinal strain, and the myocardial performance index in pediatric oncology patients. It is assumed that these novel echocardiographic measures can detect early changes in cardiac function that traditional methods cannot. Moreover, these early changes might predict long-term cardiac outcomes in this population. Accordingly, the objectives of this study are to evaluate the impact of cancer type and treatment protocol on cardiac function in pediatric oncology patients; to compare the sensitivity of STE, GLS, and MPI with that of traditional echocardiographic methods for detecting early cardiac dysfunction; and to explore the potential of these measures to predict long-term cardiac complications in this population.

## 2. Materials and Methods

### 2.1. Design and Ethics

This study was designed as a single-center, retrospective cohort study conducted over a four-year period from 2019 to 2022. The study protocol and ethical considerations were reviewed and approved by an institutional review board (IRB). All study procedures complied with the ethical standards of the 1964 Helsinki declaration and its later amendments. The investigators ensured that confidentiality was maintained, and data privacy was protected according to local data protection laws. The investigators clarified to the parents/guardians that participation was voluntary and that they could withdraw from the study at any time without affecting the child’s medical care. Before participation, written informed consent was obtained from the parents or guardians of all pediatric patients. For patients who reached the age of consent during the course of the study, assent was sought in addition to parental consent. The research team ensured that all participants and their guardians fully understood the study’s objectives, procedures, potential benefits, and risks.

Studies have consistently highlighted the potential cardiotoxic effects of oncology treatments, especially in pediatric patients. While our research primarily focuses on a specific patient cohort, it is imperative to contextualize our findings within the broader scope of evidence. Several meta-analyses have aggregated results from multiple studies, providing a comprehensive understanding of cardiac effects in pediatric oncology patients across diverse treatment protocols, cancer stages, and demographics. Such meta-analyses underscore the importance of regular cardiac monitoring, especially considering the multifaceted nature of oncology treatments and the variabilities in patient response. By delving deep into the oncological characteristics of our population, such as clinical stage, total dose of cardiotoxic drug administered, and detailed echocardiogram timelines, our study aims to bridge the gaps in knowledge and offer nuanced insights into pediatric cardiac care in oncology settings.

### 2.2. Inclusion and Exclusion Criteria

The inclusion criteria were pediatric patients diagnosed with a new oncological pathology requiring anthracycline therapy between 2019 and 2022. Baseline measurements were taken upon the patient’s first presentation at the oncology ward, before initiation of any therapeutic interventions. Patients, both neoadjuvant and metastatic, were included to provide a comprehensive view of the impact of different cancer stages on cardiac function. The study focused primarily on the diagnosis of a new oncological pathology and did not differentiate based on the specific stage of cancer. However, the clinical stage of each patient was documented and will be presented in the Results section for a more detailed understanding. The inclusion criteria comprised: (1) Pediatric patients (aged 0–18 years) diagnosed with a new oncological pathology requiring anthracycline therapy between 2019 and 2023; (2) patients with measurable cardiac function at baseline and at regular intervals during the course of treatment; (3) patients whose clinical and treatment data, including disease type, treatment protocol, and cumulative dosage of anthracyclines and other chemotherapy agents, were available and complete.

On the contrary, the exclusion criteria comprised: (1) Patients with known pre-existing cardiac pathology that could affect the cardiac function measurements; (2) patients who had already started oncological treatment before baseline measurements could be established; (3) patients with any contraindications to echocardiography, such as severe skin condition or chest deformity; (4) patients who initiated treatment at our institution but decided to continue treatment at another facility; and (5) patients with other concurrent severe medical conditions (e.g., severe infections, metabolic diseases, genetic syndromes, etc.) that could independently impact cardiac function.

The control group consisted of healthy children admitted to our hospital for minor conditions such as chest wall pain, syncope spells, minor respiratory infections, or athletes who came in for their mandatory annual check-ups and required a cardiology assessment. The cases and control groups were matched by age, gender, and body mass index. The control group included healthy children admitted to our hospital for various minor conditions. Among these, some were athletes. For the purpose of this study, “athletes” were defined as individuals who engage in regular, structured physical training for a specific sport and participate in competitive events related to that sport at least once a year. Out of all the controls, seven were athletes.

Additional factors including patient age, sex, disease, fractional shortening (FS), left-ventricular global longitudinal strain (GLS), Simpson’s method of discs (SMOD) measurements, myocardial performance index (MPI), presence of cardiotoxicity, 12-lead electrocardiogram (ECG) findings, echocardiography results, biomarkers of cardiac injury, protocol intensity, surface area (m^2^), total anthracycline dose, cumulative dosage of other chemotherapy agents (including cytarabine administered intravenously, subcutaneously, and intrathecally, etoposide, cyclophosphamide, vincristine, asparaginase, ifosfamide, methotrexate administered intravenously and intrathecally, mercaptopurine orally, Oncaspar intravenously, mitoxantrone intravenously), and radiation therapy received were collected and analyzed.

### 2.3. Materials Used and Definitions

The diagnosis of cardiac toxicity was made by identifying ultrasound alterations after chemotherapy. Cardiac function was measured using a GE VIVID E9 echocardiograph. High-quality images with ECG signals were captured in apical views (3, 4, and 2 chambers). When satisfactory images were obtained, the machine software identified the end-systolic frame and automatically traced the endocardial and epicardial borders, spanning from one end of the mitral annulus to the other. This process generated a region of interest (ROI) that encompassed the entire myocardial wall. Cardiac function measurements were taken at regular intervals post the initiation of therapy. Specifically, echocardiograms were performed every three months. Cardioxane, although not a primary chemotherapy agent under consideration, was documented if administered to any patient.

Adjustments were then made to the ROI manually, and speckle placement was corrected to select for optimal myocardial wall thickness. Speckles, which are myocardial reflectors, were tracked throughout the cardiac cycle to determine myocardial strain. Care was taken not to include the pericardium or the base of the valves to avoid a false reduction in global longitudinal strain (GLS). Decreases in GLS (less negative values, closer to 0) may indicate decreased cardiac function and potentially cardiac injury.

In addition to GLS, cardiac function was assessed using traditional methods such as M mode dimension ejection fraction (Teicholtz) and volume ejection fraction (Simpson’s method of disks, SMOD), and tissue Doppler was used to measure the myocardial performance index. The normal range for SMOD is generally considered to be between 55% and 70%, while values below 55% are usually considered indicative of reduced systolic function. Normal MPI values are generally considered to be less than 0.40, and these may vary slightly depending on whether they are measured by tissue Doppler imaging or flow Doppler imaging. Values above 0.40 indicate worsening global cardiac function. Any abnormalities that arose during treatment, such as pericardial or pleural effusion, valve regurgitations or stenoses, pulmonary hypertension, diastolic dysfunction, dilated cardiomyopathy or electrocardiographic modifications, were documented and monitored.

### 2.4. Statistical Analysis

Statistical analysis was performed using the Statistical Package for the Social Sciences (SPSS v.26). Descriptive statistics were utilized to summarize the data. Continuous variables were expressed as the mean ± standard deviation, while categorical variables were presented as frequencies and percentages. The paired *t*-test was used to compare baseline and post-treatment measurements within the same group, and the independent *t*-test was used to compare differences between the patient and control groups, while the ANOVA test was employed to compare means of more than two groups. Multivariate logistic regression was employed to identify independent predictors of chemotherapy-induced cardiotoxicity, adjusting for potential confounders. Before performing multivariate logistic regression, a univariate analysis was conducted to determine which variables were to be included in the multivariate analysis. Additionally, to assess the effectiveness of our cardiac function measurements against established standards, a predictive analysis was carried out. This involved computing predictive values, sensitivity, and specificity of our measures in relation to the gold standard. The ANOVA test was primarily utilized to compare means of cardiac function measurements at different time points, including baseline, 3 months, 6 months, and 12 months. A *p*-value of less than 0.05 was considered statistically significant. All graphs and tables were created using GraphPad Prism v.9.

## 3. Results

### 3.1. Background Data of Patients

**Figure 1:**
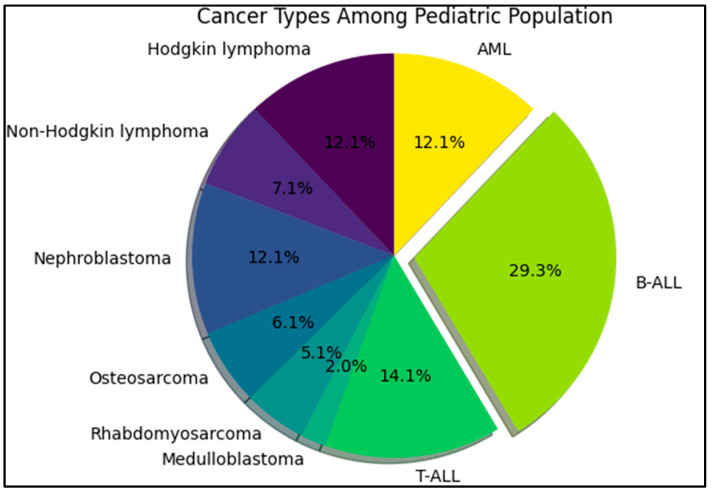
Prevalence of cancer histology types among the pediatric population.

The current study incorporated a total of 145 participants, including 99 cases and 46 controls (Table 1). The average age of the case group was 10.7 ± 4.4 years, whereas the control group averaged slightly younger at 10.2 ± 3.6 years, without a significant difference. The body mass index (BMI) was comparable between the two groups, with mean BMI values of 20.5 and 21.3 for the cases and control groups, respectively, but with no significant difference. Moreover, the BMI percentile categories displayed a balanced distribution among both case and control groups, and the statistical comparison between the two groups did not yield a significant *p*-value (0.361). Gender distribution in both groups was also similar, with 58.6% of the case group being male compared to 63.0% in the control group. Female participants accounted for 41.4% of the case group and 37.0% of the control group, yielding a *p*-value of 0.610. Therefore, gender did not significantly differ between the two groups. The most common cancer histology was B-cell acute lymphocytic leukemia (B-ALL), followed by T-ALL (14.1%), and acute myeloid leukemia (AML) in 12.1% of cases, as presented in Figure 1.

### 3.2. Cardiac Parameters

Table 2 outlines the analysis of cardiac function measurements between pediatric oncology patients post-chemotherapy (*n* = 99) and healthy controls (*n* = 46). Ejection fraction (EF), a marker of systolic function, showed significant variation between the two cohorts. The mean EF in the cases was 62.8 ± 5.7%, which was lower than that in the controls (66.4 ± 6.1%), and this difference was statistically significant (*p* < 0.001). Additionally, EF distribution across categories revealed that a higher proportion of controls had an EF above 70% (30.4%) compared to that of cases (11.1%), also statistically significant (*p* = 0.005). A similar trend was observed in the global longitudinal strain (GLS) measurement. The cases had a mean GLS of −16.3 ± 5.1, whereas the controls had a more negative mean GLS of −19.0 ± 5.4, indicating a more substantial contraction and hence better cardiac function in the controls (*p* = 0.004).

When assessed using Simpson’s method of discs (SMOD), the mean score in cases was found to be 55.1 ± 6.0, which was again significantly lower than that in controls (59.3 ± 6.7, *p* < 0.001). The myocardial performance index (MPI), a measure of both systolic and diastolic function, was higher in cases (0.37 ± 0.06) compared to that in controls (0.55 ± 0.10). A higher MPI usually represents worse cardiac performance, reflecting poorer overall cardiac function in cases (*p* < 0.001). Electrocardiogram (ECG) findings revealed a significantly higher proportion of normal findings in controls (87.0%) compared to that in cases (66.7%, *p* = 0.010). This was mirrored in the cardiac ultrasound findings, with a higher rate of normal findings among controls (76.1%) compared to that in cases (47.5%, *p* = 0.001). Conversely, the percentage of abnormal findings was higher in cases for both ECG and cardiac ultrasound.

Table 3 illustrates the comparison of cardiac function measurements stratified by cancer histology. Although there was variation among the different cancer types, none of the observed differences reached statistical significance. Global longitudinal strain (GLS) was calculated using speckle tracking echocardiography and ranged from −15.3 ± 4.6 in Hodgkin’s lymphoma patients to −18.1 ± 5.1 in T-cell acute lymphoblastic leukemia (T-ALL) patients. However, these differences were not statistically significant (*p* = 0.442).

Cardiac function measured by Simpson’s method of discs (SMOD) ranged from 51.0 ± 6.3 in acute myeloid leukemia (AML) patients to 59.2 ± 6.6 in non-Hodgkin’s lymphoma patients. Again, the differences across the various types of cancers did not attain statistical significance (*p* = 0.150). The myocardial performance index (MPI), a combined measure of both systolic and diastolic function, varied between 0.36 ± 0.05 in Hodgkin’s lymphoma patients and 0.48 ± 0.09 in medulloblastoma patients, but these variations were not statistically significant (*p* = 0.306). Lastly, mean ejection fraction (EF), a measure of systolic function, varied between 58.0 ± 5.4 in osteosarcoma patients and 64.6 ± 5.5 in non-Hodgkin’s lymphoma patients. Despite this range, the differences were not statistically significant (*p* = 0.084).

Table 4 illustrates the cardiac function measurements in relation to the different treatment protocols implemented for the various types of cancer. Despite observable differences in the measurements, none of the variations among the treatments reached statistical significance. Global longitudinal strain (GLS), calculated using speckle tracking echocardiography, varied across treatment protocols from −16.5 ± 4.6 under the EURAMOS protocol to −15.0 ± 4.8 under the RCHOP protocol. However, these variations did not achieve statistical significance (*p* = 0.095). Regarding the Simpson’s method of discs (SMOD), values ranged from 55.0 ± 6.6 under the EURAMOS protocol to 60.1 ± 4.3 under the ICE protocol, and this range did not reach statistical significance (*p* = 0.317).

The myocardial performance index (MPI), a combined measure of both systolic and diastolic function, varied between 0.39 ± 0.04 under the AML BFM protocol and 0.46 ± 0.12 under the ICE protocol, but these differences were not statistically significant (*p* = 0.063). Ejection fraction (EF), a measure of systolic function, ranged from 57.6 ± 3.9 under the EURAMOS protocol to 62.6 ± 3.8 under the ISPO protocol, but these differences were not statistically significant (*p* = 0.139). The percentage of patients who developed cardiac toxicity also varied by treatment protocol, with the highest occurrence in patients under the ALL BFM protocol (12.1%) and the lowest in multiple protocols, including AML BFM, ICE, ISPO, and RCHOP, each at 2.0%. However, the variance in incidence of cardiac toxicity among the different treatment protocols did not reach statistical significance (*p* = 0.247).

### 3.3. Risk Evaluation

**Figure 2:**
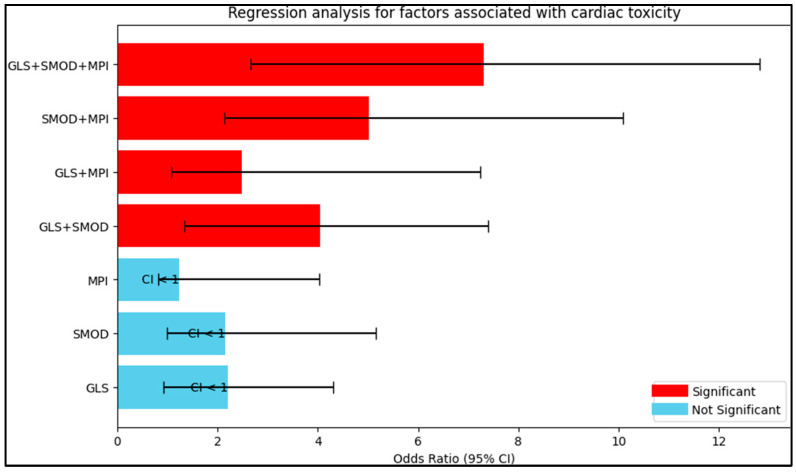
Risk analysis for factors associated with cardiac toxicity.

Table 5 and Figure 2 show the results of the regression analysis, providing odds ratios for the associations between various cardiac function measurements and the development of cardiac toxicity. The odds ratios have been adjusted for potential confounders. Global longitudinal strain (GLS) alone had an odds ratio of 2.20, indicating that for each unit increase in GLS, the odds of cardiac toxicity were approximately doubled. However, this finding was not statistically significant (95% CI: 0.92–4.31, *p* = 0.106). Simpson’s method of discs (SMOD) had a similar odds ratio of 2.16, indicating a slightly more than double increase in the odds of cardiac toxicity for each unit increase in the SMOD measure, but again, this finding was not statistically significant (95% CI: 0.99–5.16, *p* = 0.098).

The myocardial performance index (MPI) showed a less marked association, with an odds ratio of 1.24 suggesting only a slight increase in the odds of cardiac toxicity for each unit increase in MPI. This association was also not statistically significant (95% CI: 0.81–4.03, *p* = 0.221). However, combinations of these measures showed stronger and statistically significant associations with cardiac toxicity. The combination of GLS and SMOD had an odds ratio of 4.05, showing a significant quadrupling in the odds of cardiac toxicity (95% CI: 1.33–7.40, *p* < 0.001). Similarly, the combination of GLS and MPI showed a significant increase in the odds of cardiac toxicity, with an odds ratio of 2.49 (95% CI: 1.08–7.24, *p* = 0.030). The combination of SMOD and MPI showed an even greater odds ratio of 5.02, indicating a more than fivefold increase in the odds of cardiac toxicity (95% CI: 2.14–10.09, *p* < 0.001). The combination of all three measurements—GLS, SMOD, and MPI—showed the greatest odds ratio of 7.30, indicating more than a sevenfold increase in the odds of cardiac toxicity (95% CI: 2.65–12.81, *p* < 0.001).

## 4. Discussion

### 4.1. Literature and Current Study Findings

The present study’s findings underscored a significant impact of disease type and treatment intensity on cardiac function in pediatric oncology patients. These results were consistent with the initial hypotheses that traditional echocardiographic methods might not detect early changes in cardiac function that these novel methods can discern. Importantly, these early changes might predict long-term cardiac outcomes in this population, lending further weight to the potential utility of these novel approaches in this clinical context.

The observed differences in cardiac function parameters, such as ejection fraction (EF), global longitudinal strain (GLS), the myocardial performance index (MPI), and results from Simpson’s method of discs (SMOD), underscored the potential deleterious effects of cancer and its treatment on cardiac function. These findings align with earlier studies, such as Lipshultz et al. [20], which identified a higher risk of cardiac dysfunction in childhood cancer survivors, particularly in those exposed to anthracycline therapy or chest radiation.

Importantly, our study revealed no statistically significant differences in cardiac function parameters when stratified by cancer histology, suggesting a consistent impact across various cancer types. While this seems to be in contrast to prior research such as that by Mulrooney et al. [21] and Getz et al. [22], it is crucial to consider several factors. First, the chemotherapeutic agents used in those studies were different from those in ours, with some using agents like dexrazoxane instead of doxorubicin for AML. The cardiotoxic profile of different chemotherapy agents can vary widely, potentially accounting for the disparate findings. Additionally, population characteristics, such as the age group, baseline health status, and the presence of other comorbidities, might have been different in these studies, leading to varied results. Moreover, sample size, methodology, and the instruments used to measure cardiac function can also influence findings. For instance, if Mulrooney et al. [21] had a larger sample size, they might have had more statistical power to detect subtle differences that our study could not. Similarly, variations in methodology, such as the time intervals between chemotherapy sessions and cardiac function measurements, can yield different results.

The uniform impact of treatment intensity on cardiac function across various protocols, as observed in our study, deviates from previous literature. Studies like Armenian et al. [23] have indicated differential impacts based on treatment protocols. Several reasons could account for this discrepancy. The definition and classification of treatment intensity might differ between studies. What was considered “intense” in our study could potentially be “moderate” or “mild” in another. Furthermore, the follow-up duration and intervals to check cardiac function post-treatment could vary, leading to differences in the observed impacts. Lastly, the previous studies might have included more diverse treatment protocols, or their patient population might have had different vulnerabilities to certain treatments. However, it is possible that the robustness and sensitivity of the novel echocardiographic measures used in the present study allowed for a more nuanced and sensitive detection of early cardiac dysfunction, thus capturing changes that traditional methods might have missed.

Interestingly, the regression analysis results showed a significant association between combinations of novel echocardiographic measures (GLS, SMOD, and MPI) and the development of cardiac toxicity. This finding suggests that the combined use of these novel measures might provide more accurate and early detection of cardiac dysfunction in pediatric oncology patients. This supports the contention of several previous studies, such as Thavendiranathan et al. [24] and Poterucha et al. [25], who argued for the combined use of novel echocardiographic measures to improve the prediction of chemotherapy-related cardiac dysfunction. Moreover, this combined approach may have the potential to facilitate early intervention, thus mitigating the risk of long-term cardiac complications in this vulnerable population. This aligns with recent research by Narayan et al. [26], who demonstrated that early detection and intervention can improve cardiac outcomes in pediatric oncology patients.

Our results accentuate that pediatric oncology patients post-chemotherapy demonstrated significant decrements in cardiac function when compared to healthy controls. Parameters such as EF and GLS showcased notably lower values in the cases than in controls, indicative of reduced systolic function and compromised cardiac health, respectively. These findings align with the prevailing literature suggesting potential cardiotoxic effects of certain chemotherapy agents on pediatric patients, warranting heightened surveillance and potential interventions to ameliorate these impacts [27,28]. Additionally, while variations in cardiac measurements based on cancer histology and treatment protocol were noted, these were not statistically significant. This underlines the need for more expansive studies, possibly integrating multi-center data, to discern the nuanced effects of different treatment modalities and disease types on pediatric cardiac health. As childhood cancer survivors continue to live longer due to advances in therapy, understanding these cardiac repercussions becomes paramount for tailoring long-term care and optimizing quality of life.

The findings of this study also highlight the potential predictive value of these novel echocardiographic measures in forecasting long-term cardiac outcomes in pediatric oncology patients. This echoes the findings of studies such as Cheung et al. [29] and Kang et al. [30], which identified a significant correlation between early changes in these measures and long-term cardiac outcomes. Therefore, these results underscore the significant impact of cancer and its treatment on cardiac function in pediatric oncology patients. The present study’s use of novel echocardiographic measures (GLS, SMOD, and MPI) offers a valuable approach to detecting early cardiac dysfunction and potentially forecasting long-term cardiac outcomes in this population. These findings underscore the importance of ongoing research in this area to further refine these methods and to develop targeted interventions to mitigate the cardiac risks associated with pediatric oncology treatment. Lastly, there is a need for a follow-up study that looks into the time of initial detection of cardiac function abnormalities by the different echocardiographic methods, spanning a period longer than four years. This would help in drawing a more definitive comparison between traditional and novel measures in predicting anthracycline-induced cardiotoxicity.

### 4.2. Study Limitations

While this study provides valuable insights into the potential of speckle tracking echocardiography, global longitudinal strain, and the myocardial performance index as sensitive indicators of early anthracycline-induced cardiotoxicity, it has several limitations. Firstly, the study’s single-center, retrospective design might limit the generalizability of its findings. Data from a single institution might not adequately capture the variability of clinical practices across different centers, potentially biasing the results. Furthermore, the retrospective nature of the study might introduce selection bias, with the potential for missing or incomplete data. The reliance on historical records could also lead to information bias due to inaccuracies in documentation. Secondly, the study was conducted over a four-year period, which might be insufficient to determine the long-term cardiac effects of anthracycline therapy. Late-onset cardiotoxicity can occur many years after treatment cessation; thus, the follow-up period might need to be extended to fully assess these effects.

Additionally, the study did not account for other factors that might influence cardiac function, such as patients’ physical activity levels, nutritional status, or other comorbidities. The impact of these confounding variables on cardiac function should be considered in future studies. The exclusion of patients who had started treatment before baseline measurements could be established might have resulted in a selection bias, potentially excluding patients with more aggressive disease who needed immediate treatment. Furthermore, patients with severe infections or metabolic diseases were also excluded, potentially creating a cohort of patients in better overall health than the general population of pediatric oncology patients.

Lastly, while the study included a healthy control group, these controls were patients who sought medical care for minor conditions, possibly introducing a “healthy patient bias”. Furthermore, matching cases and controls by age, gender, and body mass index might not have controlled for all potential confounding variables. Other factors such as socioeconomic status or genetic predispositions might also influence cardiac outcomes and were not considered in this study. Despite these limitations, the study provides valuable insights into the potential use of novel echocardiographic measures to detect early cardiac dysfunction in pediatric oncology patients. Further research, particularly multicenter, prospective studies with longer follow-up periods, is needed to validate these findings and to better understand the long-term cardiac outcomes in this population.

## 5. Conclusions

The study concluded that pediatric oncology patients exhibited significant differences in cardiac function post-chemotherapy compared to that of healthy controls, as evidenced by measurements including the ejection fraction (EF), global longitudinal strain (GLS), Simpson’s method of discs (SMOD), and the myocardial performance index (MPI). These measurements suggested an increased prevalence of cardiac dysfunction in patients, corroborating the hypothesis that cancer type and treatment intensity significantly impacted cardiac function. Notably, early changes detected by GLS, SMOD, and MPI were associated with long-term cardiac outcomes, establishing these methods as potentially superior to traditional echocardiographic measures in identifying early cardiac dysfunction. However, the variations in cardiac function measurements among different cancer histologies and treatment protocols did not reach statistical significance. Interestingly, the combination of GLS, SMOD, and MPI demonstrated the highest association with the development of cardiac toxicity, indicating a sevenfold increase in risk. While the data indicated a promise of these novel measures, further longitudinal studies, spanning longer durations, are needed to truly validate their long-term predictive value.

## Figures and Tables

**Table 1:** Background data of study participants.

Variables *	Cases (*n* = 99)	Controls (*n* = 46)	*p*-Value
Age (mean ± SD)	10.7 ± 4.4	10.2 ± 3.6	0.471
Age range	1–18	1–17	–
BMI, kg/m^2^ (mean ± SD)	20.5 ± 4.6	21.3 ± 5.8	0.308
BMI percentile categories			0.361
>85%	9 (9.1%)	2 (4.3%)	
50–85%	47 (27.3%)	9 (19.6%)	
15–50%	75 (35.4%)	17 (37.0%)	
5–15%	81 (21.2%)	16 (34.8%)	
<5%	4 (7.0%)	2 (4.3%)	
Gender (*n*,%)			0.610
Male	58 (58.6%)	29 (63.0%)	
Female	41 (41.4%)	17 (37.0%)	

*—Mean and SD compared using Student’s *t*-test; BMI—body mass index; SD—standard deviation.

**Table 2:** Cardiac function measurements between children after chemotherapy and healthy controls.

Variables *	Cases (*n* = 99)	Controls (*n* = 46)	*p*-Value
EF (mean ± SD,%)	62.8 ± 5.7	66.4 ± 6.1	<0.001
EF categories (initial)			0.005
50–60%	16 (16.2%)	2 (4.3%)	
60–70%	72 (72.7%)	30 (65.2%)	
>70%	11 (11.1%)	14 (30.4%)	
GLS (mean ± SD,%)	−16.3 ± 5.1	−19.0 ± 5.4	0.004
SMOD (mean ± SD,%)	55.1 ± 6.0	59.3 ± 6.7	<0.001
MPI (mean ± SD, score)	0.37 ± 0.06	0.55 ± 0.10	<0.001
ECG (%)			
Normal findings	66 (66.7%)	40 (87.0%)	0.010
Abnormal	33 (33.3%)	6 (13.0%)	
Cardiac ultrasound (%)			0.610
Normal findings	47 (47.5%)	11 (76.1%)	0.001
Abnormal	52 (52.5%)	6 (23.9%)	

*—Mean and SD compared using Student’s *t*-test; SD—standard deviation; GLS—global longitudinal strain; SMOD—Simpson’s method of discs; MPI—myocardial performance index; ECG—electrocardiogram; EF—ejection fraction.

**Table 3:** Cardiac measurements based on cancer histology.

Variables (Mean ± SD)	Speckle (GLS, %)	Simpson (SMOD, %)	MPI Score	EF (%)
Cancer histology				
Hodgkin’s lymphoma	−15.3 ± 4.6	58.4 ± 6.8	0.36 ± 0.05	62.0 ± 5.9
Non-Hodgkin’s lymphoma	−16.2 ± 5.8	59.2 ± 6.6	0.41 ± 0.07	64.6 ± 5.5
Nephroblastoma	−16.0 ± 4.2	54.6 ± 5.1	0.40 ± 0.04	59.3 ± 5.1
Osteosarcoma	−15.3 ± 4.7	52.1 ± 5.3	0.44 ± 0.10	58.0 ± 5.4
Rhabdomyosarcoma	−15.9 ± 5.3	55.0 ± 6.2	0.42 ± 0.06	62.4 ± 5.8
Medulloblastoma	−17.6 ± 4.8	52.8 ± 5.6	0.48 ± 0.09	60.8 ± 5.6
T-ALL	−18.1 ± 5.1	53.3 ± 6.1	0.40 ± 0.05	61.1 ± 4.7
B-ALL	−17.3 ± 4.9	54.9 ± 5.4	0.45 ± 0.12	59.3 ± 5.5
AML	−16.4 ± 5.2	51.0 ± 6.3	0.41 ± 0.07	60.6 ± 4.9
*p*-value	0.442	0.150	0.306	0.084

SD—standard deviation; MPI—myocardial performance index; T-ALL—T-cell acute lymphoblastic leukemia; B-ALL—B-cell acute lymphoblastic leukemia; AML—acute myeloid leukemia; GLS—global longitudinal strain; SMOD—Simpson’s method of discs; EF—ejection fraction.

**Table 4:** Cardiac measurements based on treatment protocol.

Variables (Mean ± SD)	Speckle (GLS, %)	Simpson (SMOD, %)	MPI Score	EF (%)	Developed Cardiac Toxicity (*n*,%)
Treatment protocol					
AML BFM	−16.1 ± 4.3	56.2 ± 4.4	0.39 ± 0.04	58.3 ± 3.5	2 (2.0%)
ALL BFM	−15.5 ± 5.4	57.0 ± 4.3	0.44 ± 0.06	61.5 ± 4.3	12 (12.1%)
ABVD	−16.2 ± 3.5	57.9 ± 5.2	0.42 ± 0.10	59.6 ± 3.7	4 (4.0%)
ICE	−15.8 ± 6.6	60.1 ± 4.3	0.46 ± 0.12	60.4 ± 4.0	2 (2.0%)
ISPO	−16.3 ± 3.7	59.3 ± 6.4	0.43 ± 0.09	62.6 ± 3.8	2 (2.0%)
RCHOP	−15.0 ± 4.8	57.6 ± 4.5	0.40 ± 0.05	60.1 ± 4.7	2 (2.0%)
EURAMOS	−16.5 ± 4.6	55.0 ± 6.6	0.42 ± 0.10	57.6 ± 3.9	3 (3.0%)
CWS	−15.9 ± 5.4	56.8 ± 5.7	0.44 ± 0.16	59.0 ± 3.6	3 (3.0%)
*p*-value	0.095	0.317	0.063	0.139	0.247

SD—standard deviation; GLS—global longitudinal strain; SMOD—Simpson’s method of discs; MPI—myocardial performance index; AML—acute myeloid leukemia; ALL—acute lymphoid leukemia; BFM—Berlin-Frankfurt-Münster; ABVD (for Hodgkin’s lymphoma)—Adriamycin (doxorubicin), bleomycin, vinblastine, and dacarbazine; ICE—ifosfamide, carboplatin, and etoposide; ISPO (for nephroblastoma)—International Society of Pediatric Oncology; RCHOP (for non-Hodgkin’s lymphoma)—rituximab, cyclophosphamide, hydroxydaunorubicin (doxorubicin), Oncovin (vincristine), and prednisone; EURAMOS—European and American Osteosarcoma Study Group; CWS (for rhabdomyosarcoma)—Cooperative Weichteilsarkom Studiengruppe; EF—ejection fraction.

**Table 5:** Regression analysis for factors associated with cardiac toxicity.

Adjusted Factors *	Odds Ratio	(95% CI)	*p*-Value
GLS	2.20	0.92–4.31	0.106
SMOD	2.16	0.99–5.16	0.098
MPI	1.24	0.81–4.03	0.221
GLS + SMOD	4.05	1.33–7.40	<0.001
GLS + MPI	2.49	1.08–7.24	0.030
SMOD + MPI	5.02	2.14–10.09	<0.001
GLS + SMOD + MPI	7.30	2.65–12.81	<0.001

*—The control group serves as reference; CI—confidence interval; GLS—global longitudinal strain; SMOD—Simpson’s method of discs; MPI—myocardial performance index.

## Data Availability

Data available on request.
